# Parenting Style and Social Media: Impact on Children’s Dietary Patterns

**DOI:** 10.3390/nu17203254

**Published:** 2025-10-16

**Authors:** Angelica Dessì, Silvia Petza, Alice Di Carlo, Federica Infantino, Federica Zanco, Lucrezia Galimberti, Vassilios Fanos, Alice Bosco

**Affiliations:** Neonatal Intensive Care Unit, AOU Cagliari, Department of Surgical Sciences, University of Cagliari, 09124 Cagliari, Italy

**Keywords:** children’s eating behaviors, parenting style, parental feeding practice, pediatric nutrition, social media, complementary feeding, eating disorders

## Abstract

Background/Aim: Nutrition plays a pivotal role in development, not only in the early stages of life but also during adolescence, a period marked by vulnerability to the onset of overweight, obesity and eating disorders, with repercussions for reproductive and bone health. This narrative review aims to explore how parenting style, parental feeding practices and social media exposure influence eating behaviors in children and adolescents. Methods: A narrative review of the literature was performed through the PubMed and Scopus databases, including studies on participants aged 0–18 years. Both observational and interventional studies focusing on parenting approaches and the impact of social media on dietary behaviors were included. Given the narrative design, studies were selected based on conceptual relevance rather than formal inclusion or exclusion criteria, and on their contribution to understanding the multifactorial determinants of eating behavior. Results: A substantial body of research has demonstrated the critical influence of the family in cultivating positive eating habits and fostering a healthy relationship with food in children, serving as a role model and through responsive and authoritative parenting. Conversely, controlling or restrictive styles may contribute to dysfunctional eating patterns. Social media can positively and negatively influence children’s eating behaviors and parental feeding practices, promoting nutritional awareness or exposure to unhealthy food marketing and unrealistic body ideals. Conclusions: Healthcare professionals should promote an educational, trust-based approach to nutrition, empowering parents and youth for responsible digital engagement. Integrating family, school and media education is essential for preventing obesity and eating disorders in the digital age.

## 1. Introduction

Pediatric nutrition and related disorders are now a crucial field of research given their potential consequences, both immediate and future, on health [1]. There is now robust scientific evidence supporting the importance of proper nutrition from the earliest stages of development, i.e., the first thousand days of life (from conception to two years of age) [2,3]. This is essential for implementing truly effective preventive strategies against non-communicable diseases (NCDs). In fact, during this delicate period of development, characterized by rapid tissue growth, the interaction between genetic and epigenetic factors can have a significant impact on the phenotype, with important adaptive changes to the environment and consequent implications for health [2]. In addition, the latest scientific evidence has highlighted the presence of other crucial factors, such as the importance of the first thousand days of life in shaping children’s taste preferences and the positive impact of non-prescriptive approaches to establishing a healthy relationship with food, both of which are strongly influenced by parenting style [3,4,5,6,7,8]. Specifically, parental feeding styles, such as the attitudes and behavior that parents adopt to guide their children’s eating, play a crucial role in shaping early food preferences and self-regulation [5,7,8].

This is even more important when you think about the current rise in obesity and eating disorders (EDs) in both kids and teens [9]. In fact, the data on this issue are alarming: UNICEF estimates for 2022 report that 37 million children under the age of 5 and 65 million girls and 94 million boys between the ages of 5 and 19 are overweight or obese [10]. Even more recent data from the NCD Risk Factor Collaboration (NCD-RisC) estimate that globally more than one billion people are obese, including 159 million children and adolescents aged between 5 and 19 [9]. Equally worrying are the data on EDs, with recent estimates of the prevalence of anorexia nervosa and binge eating disorder (BED) reaching 6% in girls [11]. The results of a systematic review and meta-analysis in 2023 [12] highlight the presence of EDs in 22% of children and adolescents, confirming a higher frequency in females. The average age of onset is around 12 years, and attention should also be paid to the increase in the frequency of disorders with increasing BMI [12]. In fact, although recent evidence has shown that weight loss interventions are effective in improving BED and loss of appetite control (LOC) in both children and adolescents [13], other studies have found conflicting results [14,15]. Previous publications had observed that in young adolescents, restrictive diets and weight control behaviors were associated with a higher risk of developing eating disorders in later years, as well as greater weight gain [14,15]. Today, it is necessary to consider an additional complicating factor, namely the widespread use of social media, even among younger age groups. In fact, an analysis of 50 studies conducted in 17 countries has shown that social media use is associated with body image concerns, eating disorders, and poor mental health in young people aged 10 to 24 [16]. Furthermore, parents also strongly influence their children’s vulnerability to social media-related eating disorders. This occurs in various manners, including the role of parents as models, dialogue, and emotional support.

This narrative review provides an updated synthesis of the literature on how parenting style, parental feeding practices, and social media exposure shape children’s and adolescents’ eating behaviors. A non-systematic search was conducted in PubMed and Scopus, including English-language studies involving participants aged 0–18 years. Both observational and interventional studies, as well as reviews and position papers relevant to pediatric nutrition and psychosocial development, were considered. Given the narrative nature of this review, studies were selected for their conceptual relevance and contribution to understanding the multifactorial determinants of eating behavior across neurobiological, psychological, educational and digital domains.

## 2. Cycle of Nutrition: Energy Intake Control

**Figure 1:**
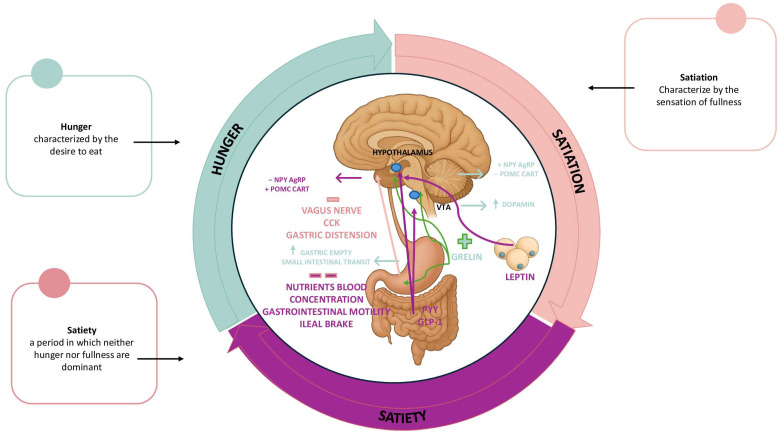
Cycle of nutrition.

In humans, the regulation of food intake is a fundamental process for survival, as it aims to maintain energy homeostasis [17,18,19,20]. This process is finely regulated by the integration of homeostatic (hormonal, neural, and metabolic) and hedonic (behavioral and cognitive) signals [18] that interact throughout the process, regulating what, when, and how much we eat [19,20]. This process consists of a hunger phase, i.e., the desire to eat, followed by a phase of acute satiety, and a third phase, prolonged satiety, characterized by a state of satisfaction that regulates subsequent food intake (Figure 1).

The first phase, hunger, is influenced by both central and peripheral factors and is triggered by real or perceived energy deficits, but can also be stimulated by environmental factors, such as sight or smell, and is responsible for the motivational phase that leads to food intake. Hunger is in fact the main trigger of the food intake process, which translates into the search for and consumption of food [19], the regulation of which is largely carried out by ghrelin, produced by cells in the gastric mucosa and small intestine, through its action on the type 1A growth hormone receptor in the hypothalamus [19,21,22]. This enhances the action of orexigenic neurons, promoting the release of both neuropeptide Y (NPY) and agouti-related neuropeptide (AgRP) and simultaneously suppressing the activity of anorexigenic neurons, reducing the release of pro-opiomelanocortin (POMC) and cocaine amphetamine-related transcript (CART) neuropeptides. At the peripheral level, this mechanism accelerates gastric emptying and intestinal transit in the small intestine [21,22]. However, ghrelin is also involved in the activation of the mesolimbic reward circuit, where it enhances the release of dopamine by neurons in the ventral tegmental area, resulting in an increase in this neurotransmitter in the *nucleus accumbens* (NA). This directly influences reward-driven behaviors, including food cravings, even in the absence of a real energy deficit [19,20,23]. It is therefore possible to distinguish between homeostatic and hedonic hunger [19,20].

After food is introduced, a phase of acute satiety begins, characterized by mechanisms that determine the size of the meal and the start of its conclusion, through peripheral and central signals. This is regulated by gastric distension signals together with oral and sensory stimuli, but also by the release of hormones by the gastrointestinal tract [17,24]. Specifically, at the peripheral level, the volume of the intestinal tract generates signals that are transmitted via the *vagus* nerve to the nucleus of the solitary tract in the hypothalamus [19]. These are joined by neuroendocrine signals, such as those mediated by cholecystokinin (CCK), which also reaches the central nervous system via the circulation [19]. At the central level, on the other hand, a cascade of molecular signals is triggered that activate the appetite-inhibiting neural system in the brainstem and hypothalamus, which undergoes further modulation by psychosocial factors [19].

Finally, food intake ends with prolonged satiety, which is responsible for appetite-suppressing effects. Regulation occurs through chemical-metabolic signals, such as blood nutrient concentrations, mechanical signals mediated by gastrointestinal motility and the ileal brake, and neuroendocrine signals through hormones such as leptin, glucagon-like peptide 1 (GLP-1), and peptide YY (PYY) [19]. Leptin, released by adipose tissue, GLP-1 and PYY secreted in the intestine, promote satiety by stimulating the anorexigenic POMC/CART neurons and inhibiting the orexigenic NPY and AgRP neurons. A further regulatory mechanism is expressed through stimulation of the *vagus* nerve. In addition, GLP-1 also extends its effects to less central areas, such as the prefrontal cortex, thalamus, and amygdala, which are responsible for the cognitive-emotional component of eating behavior. The processing and integration of all these signals by the nervous system results in slower and more prolonged inhibitory regulation [17,25,26].

## 3. Regulation and Dysregulation of Eating Behavior

**Figure 2:**
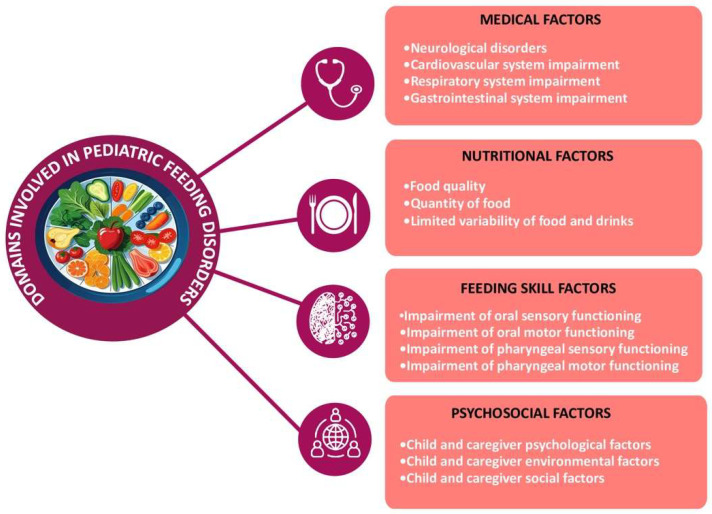
Domains involved in PFD.

The dynamic and coordinated integration of multiple physiological systems to ensure adequate food intake is an extremely complex process [27,28]. In fact, in addition to the synergistic action between the central and peripheral nervous systems to coordinate homeostatic and hedonic signals, the integration of the oropharyngeal and swallowing mechanisms is necessary, the cardiopulmonary system, the gastrointestinal tract, as well as the support of the craniofacial structures and the musculoskeletal system. Nutritional factors, motor skills related to eating, and psychosocial aspects cannot be overlooked either [27,28]. It follows that the alteration or disruption of one or more of these systems may expose the child to the risk of developing an eating disorder, with consequent physical, cognitive, emotional, and social complications [28,29,30,31], as well as increased caregiver stress [31]. In fact, as proposed by Goday et al. [28], through a multidisciplinary consensus in 2019, there is a need to assess and treat pediatric eating disorders through four complementary areas: medical, psychosocial, nutritional, and feeding skills. The concept of ‘pediatric feeding disorder’ (PFD) has been proposed, defined as an altered oral food intake that is inappropriate for age, associated with medical, nutritional, feeding skill, and/or psychosocial dysfunction (Figure 2). It is not classified unambiguously as an eating disorder and is not currently included in the DSM-V, but it can be considered a related clinical condition with its own characteristics, often associated with early neurodevelopmental, medical, or behavioral factors. Estimates indicate that this disorder is present in 25% to 50% of young children [28].

In an extremely complex context, it is therefore imperative to preserve the delicate balance that regulates food intake, and in this context, the promotion of proper eating habits from infancy is essential. In fact, it is through parents that food preferences and eating patterns are formed [8,32,33]. This can occur either through direct influence, such as controlling food intake, or indirectly, simply by acting as a positive or negative role model [32]. In the systematic review by Shloim et al. [32], the presence within the main parenting styles (authoritative, authoritarian, indulgent, and uninvolved) of a subcategory, defined as feeding style, is highlighted, which reflects the same type of need and responsiveness but applied to the food context [32]. This implies that, in the presence of an authoritative feeding style, parents actively encourage their children to eat by using supportive behaviors together with engaging explanations, as opposed to the authoritarian style, where encouragement to eat follows strict rules centered on the parent. The feeding practices that can be used depending on the style vary, but the most common include direct control through the more or less strict restriction of certain types of food or its availability in the feeding routine, pressuring children to eat, or using food as a reward or punishment, but also promoting eating patterns through example [32,33]. The importance of feeding style is supported by the main guidelines on complementary feeding, which unanimously encourage a responsive approach to feeding young children [34,35,36]. This is not limited to providing adequate amounts of micronutrients and macronutrients based on the child’s age and nutritional needs (choice of food availability/offer), but also includes planning eating habits that encourage independent food intake always and only in response to personal physiological needs of hunger and satiety, while fully respecting and promoting self-regulation in favor of adequate cognitive, emotional, and social development [34,35,36]. At the heart of this method is reciprocity between the child and caregiver during mealtimes, with the caregiver being able to recognize and respond appropriately to the child’s hunger and satiety cues to promote a positive eating experience tailored to their needs [34].

### 3.1. From Fetal Life to the First Six Months of Life

Feeding style is crucial from the earliest stages of development, throughout the first thousand days of life and beyond [3]. In this regard, breast milk not only ensures adequate nutritional intake in the first six months, representing the gold standard of nutrition for both full-term and preterm infants [37,38,39], but also provides a unique bonding experience between mother and child [37]. This bond also involves a maternal feeding style that is more responsive to the baby’s signals, given the impossibility of verifying the amount of milk consumed, which therefore requires trust in the baby [40,41]. There is also the different mechanics of sucking, which in the case of breastfed babies is active and seems to be associated with a greater capacity for self-regulation on the part of the baby compared to sucking through a baby bottle nipple, which is more passive and therefore less tiring and thus more likely to encourage overeating [41,42]. Added to this is a possible contribution mediated by early exposure to leptin present in breast milk [41]. These factors may contribute to the prevention of PFDs and add to the undisputed nutritional qualities of breast milk [43], which are very important for the prevention of childhood obesity [37,44,45], thanks also to its important effects on the intestinal microbiota [46]. There is also the hypothesis of the influence of early taste education, i.e., the relationship between the diet of mothers during pregnancy and breastfeeding and the overall food intake of children [47,48,49,50]. There is, in fact, moderate evidence to support the idea that prenatal sensory experiences can influence postnatal food preferences, facilitating the acceptance of certain flavors after birth [48,49]. This process is possible thanks to the early maturation of taste and smell receptors, which, as early as the 12th week of gestation, allow the fetus to perceive and memorize chemical stimuli from the mother’s diet [50]. This process continues during breastfeeding, as aromas from the mother’s diet appear to be transmitted to breast milk, flavoring it, although a systematic review of the literature highlights the impossibility of drawing firm conclusions to describe this relationship [47,48,49,50].

### 3.2. From Six Months to Childhood

**Figure 3:**
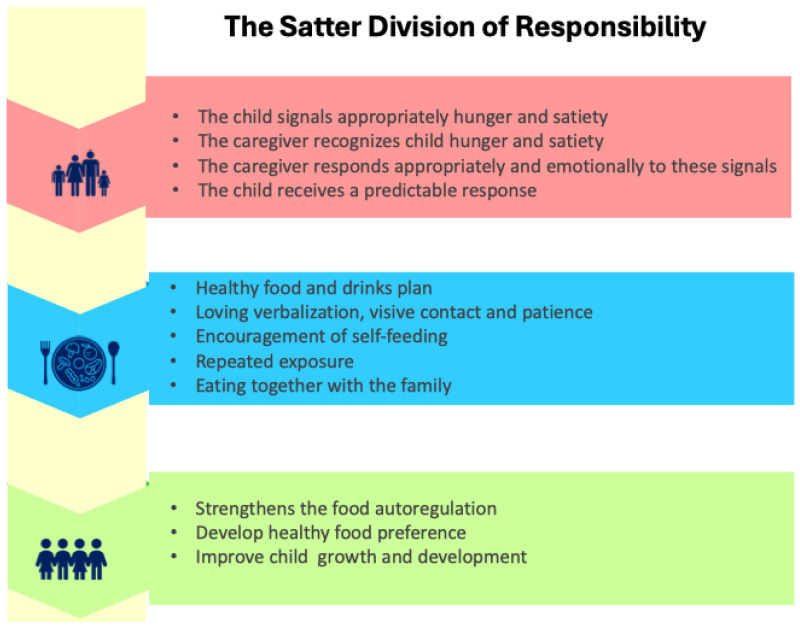
Satter Division of Responsibility.

The WHO [34], together with the recommendations on BF developed by the joint working group SIPPS (Italian Society of Preventive and Social Pediatrics), FIMP (Italian Federation of Pediatricians), SIDOHaD (Italian Society for Obesity and Eating Disorders) and SINUPE (Italian Society of Pediatric Nutrition) [36] and those of EFSA (European Food Safety Authority) [35] highlight the importance of starting to introduce solid foods around six months of age. It is also specified that for premature infants, reference should be made to the corrected age [34]. This is because there is no need and, above all, no advantage in introducing foods other than milk before this stage of development, as both breast milk and formula are adequate and sufficient to meet nutritional needs until six months of age [32,33,34,35]. Furthermore, the main recommendations emphasize that the beginning of complementary feeding should not be based solely on the achievement of adequate intestinal and renal maturation, but should take into account the child’s neurodevelopment. This implies the ability to maintain a seated position with support, to express signs of hunger and satiety, and to reach for a spoon and food. These skills are acquired around six months of age by 97% of infants [36], and even if they are achieved earlier, they do not imply the need to anticipate the introduction of solid foods [35]. Reaching this level of neuro-motor development is absolutely essential to allow the child to play an active role during meals, in line with the principles of responsive feeding unanimously supported by all guidelines [34,35,36]. These recommendations are derived from a clinical and educational precursor formulated in the 1980s by Ellyn Satter, the principle of division of responsibility outlined in Figure 3 [27,51,52].

This model was designed to establish a relationship of trust with food, in an attempt to ensure an enjoyable meal but also to prevent or treat problems related to children’s eating. The two basic principles are parental leadership in feeding and child autonomy in eating. Therefore, it is the caregiver who chooses not only which foods are suitable for the child but also ensures an appropriate emotional (where) and physical (where) environment with correct meal times (when) to promote a predictable, non-coercive but structured eating environment. It is also essential that parents demonstrate confidence in their child’s ability to determine what, how much, and whether to eat, respecting their innate capacity for self-regulation [51,52]. Thanks to these principles, a break has been made with the prescriptive view of nutrition in favor of an educational approach based on trust and nutritional competence [5,7].

The introduction of complementary foods is also fundamental in terms of taste and sensory education, marking the transition from an exclusively milk-based diet to the discovery of new flavors, smells and textures [34,53,54,55,56,57]. With regard to textures, the existing literature suggests that there is a critical period, within 10–12 months, for the acceptance of food particle sizes and the development of oral and motor skills with off-switch characteristics if exceeded. At the sensory level, it would in fact be advisable during this period to stimulate the oral cavity with a variety of textures, as lack of exposure could lead, especially in predisposed children (e.g., with tactile hypersensitivity), to increased oral sensitivity and consequent rejection of more complex textures [55]. It therefore appears that delayed introduction of lumpy foods (after 10 months) may coincide with reduced plasticity of the sensory and oral circuits involved in food acceptance. In fact, data from the Avon Longitudinal Study of Parents and Children (ALSPAC) cohort showed that delayed introduction (>9 months) of lumpy solids is associated with a higher likelihood of eating problems at age 7 (selectivity, quantity, refusal) and lower variety and consumption of fruits and vegetables [58]. Further supporting the importance of acquiring these skills is a very recent study [57] that analyzed 8851 French children. The results showed, through neurodevelopmental assessments conducted at 1, 2 and 3.5 years of age, that late introduction (after 10 months) of food in pieces can have negative extra-alimentary consequences, affecting the cognitive, linguistic and motor development of the child [57]. The delicate role of taste exposure in the formation of food preferences in children has ancient roots [59,60,61]. In fact, for evolutionary reasons, children have an innate preference for sweet, salty and umami tastes and an aversion to bitter and sour tastes. This is probably justified by the fact that in the past it was essential for the human species to ensure survival by eating energy-dense foods and avoiding potentially toxic compounds [59,60,61]. However, the context today has changed significantly, and scientific evidence has shown that taste development originates from a close correlation between genetic predisposition and environmental influences in the first thousand days of life. As a result, different eating habits arise from the food to which we are exposed, with a consequent impact on future food choices and therefore on health [34,53,54,55,56,57]. A 2020 longitudinal study [53] of 1179 children, using an objective measure of dietary quality (Youth Healthy Eating Index-YHEI) and careful statistical analysis to eliminate confounding variables, showed that different feeding practices patterns are associated with dietary quality at age 3. Specifically, it was observed that early and varied introduction of solid foods promotes the adoption of healthier eating behaviors [53]. This confirms the findings of previous literature reviews, which, based on the analysis of observational and experimental studies, concluded that early exposure to different foods, especially vegetables, facilitates food acceptance [54,55]. Very recent confirmation comes from a 2024 study [56] that analyzed 321 mother-child pairs to assess how early exposure to different foods influences dietary quality in the first years of life. The study investigated the influence of children’s appetitive traits, i.e., individual characteristics that describe how children behave when faced with food (early satiety, slow eating, avoidance, pleasure, seeking), using the Baby Eating Behavior Questionnaire (BEBQ). The study showed that dietary exposure during the first two years of life has significant effects on dietary quality up to the age of 5. Specifically, it emerged that frequent introduction of fruit and vegetables within 12 months and their regular consumption between 1 and 2 years of age is associated with better dietary quality in children at both 3.5 and 5 years of age. These benefits are even greater for children who show early satiety or slow eating. Conversely, the provision of discretionary foods such as snacks and sweets, starting at 6 months and more frequently at 2 years, is associated with poorer diet quality, regardless of the child’s appetitive traits [56]. This evidence supports the recommendations of the WHO [34], EFSA [35] and the European Society for Pediatric Gastroenterology, Hepatology, and Nutrition (ESPGHAN) [62] on complementary feeding, namely, to avoid offering foods with added sugars until 24 months of age. In fact, although only excessive sugar consumption is linked to an increased risk of obesity, diabetes and tooth decay, the main concern in children is the potential impact on the formation of food preferences. It is this exposure that can reinforce innate preferences for sweet foods, hindering the acceptance of less rewarding foods and promoting a lower dietary quality in young children, completely independently of their appetite traits [53,54,55,56,57,62].

Added to this is the scientific community’s debate on food addiction. The effect of sugary foods and more generally, ultra-processed foods (UPFs) on the brain’s reward systems is still being studied [63,64,65,66,67,68,69,70,71]. Preclinical studies have shown that sugar stimulates the mesolimbic dopaminergic system through specific neurobiological mechanisms such as synaptic plasticity, the formation of silent synapses and the modulation of certain transcriptional factors (ΔFosB/cFos and CREB, cAMP response element-binding protein) [63,64]. In animal models, this has been associated with behavioral changes such as craving and withdrawal, similar to those observed in drug use. However, many researchers argue that human evidence is still contradictory and that generalization from the animal model is not possible due to the experimental feeding conditions, which do not reflect the complex cycle of food intake in humans and do not take into account cultural, social and educational factors [65,66]. In fact, the addiction-like behaviors observed in animal models cannot be attributed solely to the type of food but also to the mode of access to that food, which is sporadic and unpredictable, reproducing a “binge” consumption that not only amplifies the hedonic value of food but also triggers excessive reactions such as craving, withdrawal, and compulsivity, which are not related to real hunger but to reward [66]. Furthermore, the levels of dopamine released following food intake are extremely lower than those related to substances of abuse [65] and the activation of brain circuits occurs with different dynamics [64,67]. Some authors have therefore proposed an alternative concept that emphasizes addiction to eating behavior as ‘eating addiction,’ in order to avoid pathologizing behaviors linked to very complex psychological and cultural contexts [68]. Critical issues regarding the concept of food addiction emerge from the work of Ziauddeen et al. [66], which highlights how problematic eating behavior does not necessarily imply a true neurobiological addiction but may derive from multiple factors (psychological, cultural, environmental). This research also shows that the hedonic pleasure associated with the consumption of certain foods is a natural and adaptive component of human nutrition and that a particular liking for sweet, salty and fatty foods does not in itself imply addiction. In fact, it is highlighted that the association between highly rewarding foods, such as sweets and salty snacks, and repeated consumption patterns is largely mediated by learning and habit, further supporting the important role of taste education. Therefore, evidence seems to show that a particular food composition can influence brain reward mechanisms, but that it is regular exposure to such foods that generates a sensory adaptation responsible for a less positive perception of more natural foods [66]. Furthermore, data from a very recent study conducted with nuclear magnetic resonance imaging, albeit in an adult population, have shown that habitual exposure to particularly palatable foods, particularly UPFs, is associated with microstructural alterations in brain regions involved in the reward system [69]. The alterations, attributable to a reduction in intracellular density and an increase in average diffusivity, suggest an impairment of brain microstructure that could influence the regulation of eating behavior and reward perception. This seems to be attributable to systemic inflammation, and these effects have been observed independently of the presence of obesity or other metabolic factors, raising the hypothesis that brain damage is related to dietary quality and not to body weight [69]. The possible role of UPFs in the dysregulation of eating behavior in children has recently been investigated in a literature review [70]. The authors highlighted how the particular composition and palatability of these foods, characterized by high levels of sugar, fat, salt, and additives, in combination with particular consumption patterns that are usually automatic and associated with a particular context (e.g., between meals, without real hunger, without awareness, and during distracting activities), may contribute, in theory, to a progressive desensitization to homeostatic signals and greater dependence on hedonic stimuli, resulting in eating behavior driven by the pursuit of gratification rather than real nutritional needs [70]. Very recent confirmation, albeit in adults, comes from a randomized, controlled, crossover clinical trial [71] that compared two isocaloric diets balanced according to nutritional guidelines, one with UPFs and one with minimally processed foods (MPFs). The results showed that even with the same nutrients, the very nature of UPFs, in terms of composition and palatability, is responsible for higher food intake, probably due to the activation of brain circuits linked to reward and a reduced sense of satiety. In fact, UPF consumption was found to be associated with lower PYY and GLP-1 release after meals, supporting reduced homeostatic satiety, along with higher ghrelin levels, suggesting that the increase in intake was not attributable to greater physiological hunger but to hedonic factors [71].

### 3.3. Adolescence

In later life, nutrition continues to play a crucial role in health, not only to ensure proper growth and the prevention of non-communicable diseases (NCDs), but also to ensure adequate sexual maturation. The role of nutrients as cofactors, precursors, and regulators of sex hormone synthesis and function is now well established, as is the close influence of nutrition on the functioning of the hypothalamic-pituitary-gonadal axis. Therefore, an alteration in the nutrition cycle during school age and even more so during adolescence can be associated not only with overweight, obesity, and EDs but also with delayed puberty or precocious puberty, with possible repercussions on fertility and bone health. Early recognition and intervention are therefore essential to limit the irreversibility of some of these effects [72,73]. Furthermore, adolescence is a crucial stage of development, comparable to the first thousand days of life in terms of obesity prevention. This is due to the important hormonal, metabolic, neurological, and behavioral changes that occur during this stage of development, which are responsible for an increased risk of overeating. Furthermore, these changes coincide with greater exposure to external stimuli, especially peer pressure, marketing, and social media, together with a decline in physical activity, often caused by giving up sports, and an increase in meals eaten alone outside the home [74]. In fact, a particular synergy between environmental and behavioral factors leads to an increase in the frequency of both obesity and EDs in adolescence [75]. In detail, from an etiological point of view, obesity is the result of the interaction between environmental, behavioral, genetic, and metabolic factors [76] responsible for the heterogeneity of phenotypes and clinical presentation [77]. Starting from school age but especially in adolescence, various environmental and behavioral factors begin to play an increasingly important role, including easy access to energy-dense foods (junk food), regular consumption of sugary drinks, portions exceeding caloric needs, and a predominantly sedentary lifestyle [78]. As for the role of genetics, in most cases obesity is polygenic [38] and only 3–5% of children have monogenic obesity [79]. With regard to EDs, disabling and sometimes fatal mental disorders, they are responsible for significant impairment of physical health and altered psychosocial functioning. Six main EDs are recognized diagnostically, with different frequencies depending on gender and age. In school age and adolescence, anorexia nervosa, bulimia nervosa, and BED are certainly more frequent, while eating avoidance and restriction disorder, pica, and rumination disorder are less frequent [80,81,82,83,84,85,86]. The etiology of EDs is also multifactorial and derives from a complex interaction between genetic, biological, environmental, psychological, familial, and sociocultural components, with variations depending on the disorder. Anorexia and bulimia appear to be closely related to particular personality traits such as perfectionism, low self-esteem, anxiety, and impulsivity, which are associated with cognitive distortions of body image and the need for control. These conditions are often associated with dysfunctional family dynamics, such as overprotection or impaired emotional communication, which not only contribute to the onset of the disorder but also to its maintenance [82]. In BED, on the other hand, impulsivity and emotional dysregulation prevail, especially when associated with obesity [38].

A further complicating factor in this particular stage of development is the bidirectional interaction between obesity and eating disorders. It is now known that obesity can increase the risk of developing eating disorders, while the latter can contribute to the maintenance or worsening of obesity, creating a vicious circle that negatively affects physical and psychological health [83].

Social pressures that can contribute to the development of unhealthy eating behaviors, which are particularly relevant at this stage of development, have increased with the advent of social media. In fact, these modern means of communication, permeated by image culture, can promote body dissatisfaction, particularly during adolescence, not only because adolescents are particularly sensitive to the “gaze of others” but also because of the important physical changes they undergo [87,88]

## 4. Child–Parent Attachment and Eating Behavior

It is now well established that human behavior depends on the interaction of various environmental factors and personal characteristics, such as genetics, gender, and age. Several studies have examined the factors that specifically influence eating behavior in children from early childhood, a period when eating habits are established that will then influence the entire course of life [8,89,90,91]. The important impact of low socioeconomic status on the adoption of unhealthy eating behaviors certainly cannot be overlooked, but parenting style plays a central role [8]. In fact, educational feeding, initially proposed by Satter and then expanded and integrated into the Eating Competence (EC) model, is the precursor to responsive feeding promoted by international guidelines [34,35,36]. This educational feeding proposes a more complex view of the act of eating, which goes far beyond the quantitative nutritional aspect, integrating behavioral, social, and biological dimensions. This implies the need to consider pleasure in food, self-regulation, body acceptance, and the establishment of stable eating routines [7,92]. The measurement and validation of this new approach to nutrition took place in subsequent years and confirmed that good nutritional competence is associated with higher quality diets, fewer dysfunctional eating behaviors, and greater metabolic and psychological well-being [5,92]. This model clearly highlighted the importance of child-centeredness, in line with an authoritative parenting style, unlike other approaches where there is a predominant authoritarian management of children’s eating by parents [5,7,27,32,33,92]. It is essential that the family system establishes and promotes healthy eating behaviors through role modeling, food choices, and support [8]. In particular, parents play a decisive role both as planners of choices and offerings and in their approach to their children’s nutrition, which defines a more or less positive model. Parental feeding practices are an important feature of the early environments in which infants and children learn about food and eating, and include not only what is given to the child, but also how, when, and in what context food is offered [89,90]. The responsiveness of parents is also important, as they must be able to interpret hunger and satiety signals correctly and respond accurately to them in order to ensure the proper development of the child’s self-regulation skills [90,91]. Therefore, parents who eat properly influence their children to eat well, especially in the early years of life, as children’s first form of learning is to observe and imitate the actions of their role models [8]. Timing is also important, as the foundations of healthy eating habits must be laid before the age of 3, which is considered a sensitive window for the development of eating habits. These are fundamental roots of the aforementioned taste education, which will then influence late childhood and early adulthood [89]. This is confirmed by data from a recent systematic review of the literature [33] that analyzed the role of parenting practices in sugar consumption and their impact on the eating behaviors of children and adolescents. Specifically, the authors found that coercive parenting styles, particularly those characterized by high control and strict restrictions on sugary foods, are frequently associated with compensatory reactions in children, with an increased desire for prohibited foods and a greater likelihood of developing dysfunctional eating behaviors such as excessive sugar seeking, emotional eating, and poor self-regulation. Conversely, autonomy-oriented parenting practices, such as active involvement of children, positive modeling by parents, and a non-coercive educational approach, are associated with healthier and more sustainable eating patterns over time. These protective effects were particularly evident when such practices were adopted early in life, confirming once again the importance of early intervention in the development of eating habits. These results have therefore demonstrated that structured practices such as the regulated availability of healthy foods and the establishment of consistent eating routines can reinforce positive dietary behaviors without resorting to strict control, suggesting the need for a balance between environmental structuring and support for self-regulation [33]. Further confirmation comes from the meta-analysis conducted by Say et al. [93], which systematically examined the link between restrictive parenting practices in the food domain and the eating behaviors of children aged 2 to 12 years. The aim of the study was to analyze how explicit or implicit restrictive parenting practices influenced specific aspects of children’s attitudes toward food. Analysis of 24 observational studies showed that overt restriction, i.e., direct and visible control over the type and amount of food consumed, was significantly associated with greater food responsiveness, emotional overeating, and food fussiness, as well as a faster pace of eating. These behavioral patterns are considered predictive of dietary dysregulation and an increased risk of developing obesity or eating disorders in childhood. Conversely, implicit practices (covert restriction), i.e., more subtle forms of restriction, such as through the organization of the home environment or selective availability of food, and more generally non-coercive practices, did not show significant correlations with negative outcomes in eating behaviors. These results demonstrate that the way in which parental control over food is exercised is crucial, although restriction as such has also been associated with negative outcomes. Indeed, studies conducted in the early 2000s had already shown that pre-adolescents and adolescents on self-imposed restrictive diets were more prone to weight gain than weight loss over time [94]. The analysis of 15,000 adolescents followed over time showed that a self-imposed restrictive dietary approach in overweight adolescents and pre-adolescents not only failed to prevent weight gain but was also associated with an almost threefold risk of becoming overweight compared to non-dieters, after adjustment for initial BMI [94]. These findings highlight the importance of adopting sustainable strategies that include physical activity without severe total calorie restriction, which, although useful for weight loss in the short term, is ineffective or even counterproductive in the long term [94]. Maternal BMI also appears to influence a mother’s attention to her child’s nutrition. A high maternal BMI has been found to correlate with a greater adoption of restrictive eating practices involving limitations or prohibitions on certain foods and/or undue restriction of food quantity [91].

The central role of the family in determining healthy behaviors in children and adolescents through the structuring of eating habits, the promotion of a healthy lifestyle, and an appropriate attitude toward food is therefore clear. In fact, a consistent picture emerges in which coercive educational practices towards food not only compromise the development of self-regulation but can also encourage harmful compensatory strategies, such as early dieting, with paradoxical effects on weight gain. Therefore, parenting practices together with eating habits are a key primary prevention target for reducing malnutrition and eating disorders [8,89,90,91,92,93,94], however despite the availability of systematic reviews and meta-analyses on the subject, the predominantly observational nature of the studies included does not allow causal relationships to be established between parenting practices and children’s eating behaviors. The available evidence describes consistent associations but does not demonstrate direct cause-and-effect relationships, requiring further longitudinal and experimental studies to clarify the directionality of these relationships.

Added to this is the role of educators, given the number of hours children spend in schools. In this regard, two researchers from the Rochester Institute of Technology (RIT) published NEEDs for Tots (NFT) in 2017, an experimental educational program based on Satter’s division of responsibilities, which promotes healthy eating and positive relationships with food by teaching adults to guide without forcing and children to eat independently, respecting internal signals of hunger and satiety in an attempt to create consistency between home and school at a crucial stage of dietary development [95]. This is in line with the need for and importance of implementing strategies for the prevention and treatment of childhood and adolescent overweight and obesity through a gradual approach to weight management using lifestyle intervention programs combined with a multidisciplinary approach that acts at different levels, including the individual, family, institutional, and environmental levels [38,39]. Within this multidisciplinary management, behavioral strategies involving both the child and the family are essential, with a view not only to reducing calorie intake but also to reducing sedentary lifestyles through lifestyle changes, which also benefit cognitive development and school performance [96,97,98,99].

## 5. Eating in the Age of Social Media: Between Perception, Representation and Reality

The emergence of new technologies, which has seen a surge since the COVID-19 pandemic, has had a profound impact on everyday life, giving rise to previously non-existent human behaviors and raising the need for scientific and ethical analysis [88].

While digital platforms play a positive role in allowing even the most fragile personalities to express themselves, they also undermine the psychological well-being of individuals, especially when used excessively and incorrectly. Social media are designed to attract and trap users, exploiting captivating algorithms and the dopamine reward system [100,101]. This is activated through scrolling (moving your finger across the smartphone screen) and leads to the association between a specific action and a feeling of well-being. In fact, studies investigating the relationship between brain activity and social media use, using neuroimaging techniques, particularly functional magnetic resonance imaging (fMRI), have shown that addiction to these platforms can activate the reward circuit, with the release of dopamine and involvement of the ventral striatum, including areas adjacent to the NA. Dopamine plays a crucial role in cognition and behavior, participating in numerous processes, including the regulation of behavioral variability in response to reward stimuli. It has also been observed that individuals who use social apps more frequently tend to have a lower capacity for dopaminergic synthesis, a condition that could contribute to greater vulnerability to compulsive seeking of rewarding stimuli [100,101,102]. A further mechanism through which social media keeps users ‘glued’ is the unpredictability of possible rewards, with the consequent frantic search for the next dopamine release [101]. Given the critical nature of adolescence for neurocognitive development, in recent years, several studies have sought to more precisely delineate the association between habitual social media use and brain development trajectories, both from a functional and structural point of view, in this population. This need arises not only because childhood, but especially adolescence, is a crucial period for the profound changes affecting the maturation of the nervous system, which is extremely plastic and therefore more susceptible to external insults, but also because of the amount of time spent on social media and digital platforms: 5.44 h per day according to recent estimates [88,103,104].

Evidence suggests that prolonged and repetitive exposure to social media during adolescence can alter the physiological trajectory of brain development, particularly at the functional level (connectivity between cognitive and motivational networks) and, to a lesser extent, at the structural level. These changes seem to reflect a greater sensitivity to social rewards and digital environmental cues, with potential consequences for behavior, mood, and emotional regulation in young people [105,106,107,108]. Specifically, a longitudinal study [105] using MRI found that greater daily social media use is associated with slower growth in cortical thickness in frontal regions. These areas, which are involved in cognitive control, undergo changes consistent with an impact on the natural neuroanatomical development of adolescence, although no direct reductions in ventral striatum volume have emerged [105]. Similar research has observed that individuals with habitual social media ‘checking’ behaviors (frequent, automatic, and repetitive use) show greater growth, compared to their peers with less frequent use, in the functional connectivity between specific neural networks involved in attention regulation, social signal evaluation, and decision-making and motivational processes, suggesting greater sensitivity to social stimuli and reward in young habitual users [106]. Another large longitudinal study [107] that analyzed pre-adolescents (aged 9 and 11) to assess how long-term digital media use affects brain development found no changes in total or cortical brain volume. However, a small, statistically significant sex-specific variation in cerebellar volume was found: a decrease in males and an increase in females [107]. This could suggest a possible sex-specific vulnerability of cerebellar development to ongoing digital exposure. However, the main findings of the Adolescent Brain Cognitive Development (ABCD) Study, the largest longitudinal study ever conducted on neurocognitive development in childhood, documented the absence of robust scientific evidence regarding the correlation between moderate use of digital media and structural damage to the brain. On the other hand, excessive use, in terms of hours per day or at night (before bedtime), has been linked to changes in the prefrontal cortex, which is involved in inhibitory control and emotional regulation, to reduced sleep quality and duration, and to increased psychological distress and depressive symptoms [108]. In this regard, the scientific community is developing a consensus definition of the concept of problematic social media use (PSMU) [88]. From a psychosocial perspective, PSMU has been observed to cause changes in eating habits and biological functions, affecting neurotransmitter regulation and circadian rhythms [88]. The consequences are also reflected at the physical level, with disorders such as cervical stiffness (tech neck), tendinopathy of the first finger of the hand (texting thumb), and an increase in the incidence of tooth decay due to poor eating habits [109]. In addition, excessive use of digital devices affects cognitive and psychological aspects, promoting attention disorders, mood swings, and anxiety, but it also has social implications. It is responsible for a reduction in perceived quality of life [88,102]. Adolescents often seek compulsive smartphone use to cope with boredom, which is perceived as an unpleasant and difficult-to-overcome emotional state [109]. The main warning signs for PSMU are loss of control, mood changes and withdrawal-like symptoms. For these reasons, in order to detect PSMU early and identify its prevalence, the Bergen Social Media Addiction Scale, the Social Media Disorder Scale [102], and the Digital Addiction Scale for Children (for children aged 9 to 12) [109] have been developed for screening purposes.

The problems associated with social media use by children and adolescents are not limited to the risk of impaired neurological maturation or the development of PSMUs, but also include numerous other threats. In fact, the critical issues range from cyberbullying to poor sleep hygiene, also affecting the possibility of developing excessive attention to body image, eating disorders, or obsessive eating practices (such as orthorexia) [102]. The most recent research has identified the relationship between social media use, body image, and EDs as a key area of investigation [16,88,108,109,110,111]. This stems from the fact that, with the advent of social media, teenagers are catapulted into an aesthetic reality in which the display of defined and proportionate bodies, often not real but created through the use of filters, is normalized as part of a process of social integration. Confronting this reality with an identity that is still structurally fragile can lead to exacerbated dissatisfaction with one’s body image [108], a dynamic observed in both sexes [88]. A further alarming factor is the support for EDs on social media, although the most popular platforms have tried to address the problem by flagging and blocking certain content [88]. A recent scoping review that analyzed 50 studies published between 2016 and 2021 on adolescents and young adults (aged 10–24) found that social media use is strongly associated with body image concerns, clinical and subclinical eating disorders, and mental distress [16]. Among the main mediators highlighted are social comparison, internalization of the thin/fit ideal, and self-objectification, i.e., viewing one’s body as if it were “for others” and no longer experienced as part of oneself. Pro-EDs content, the use of filters and image-focused digital platforms such as Instagram and TikTok have been confirmed as significant risk factors. Female gender and high BMI have also been highlighted as moderators responsible for an increased risk, while body appreciation has been found to be protective [16]. Although these results do not come from a systematic review of the literature and most of the studies analyzed were cross-sectional, and therefore unable to demonstrate direct causality, the associations that emerged raise concerns. Further concerns about the relationship between screen time and early symptoms of EDs emerge from the analysis of data from a large cohort of pre-adolescents in the ABCD study [110]. It was found that more daily screen time was associated with more eating disorder symptoms, including fear of gaining weight, food restriction, skipping meals, and body dissatisfaction. Specifically, each additional hour of screen time, including social media, video games, TV, messaging and video chat, was associated with an increase in the risk of BED symptoms from 5% to 55%, depending on the type of activity. Meanwhile, problematic screen use, measured using the Problematic Media Use Measure-Short Form, was found to be associated with an increased risk of between 26% and 82% of experiencing one or more related DCA symptoms, such as obsessive thoughts about weight, skipping meals, or adopting calorie restrictions [110].

Likewise, the role of the family is fundamental, as effective communication and active parental supervision can be an important protective factor against the absorption of unrealistic and harmful models. Furthermore, the family context is an important mediator in the relationship between exposure to social media and the development of psychopathological symptoms, supporting the idea that a stable emotional network and relational support can mitigate the negative impact of digital content, including unreliable nutritional information and the promotion of highly restrictive or extreme eating behaviors, on vulnerable minors [88,109,110]. Similarly, but with the opposite effect, social media use can encourage junk food consumption and promote uncontrolled eating behaviors that can lead to overweight and obesity. In fact, early use of social media (starting in primary school) and daily use of two hours or more per day have been found to be associated with an increase in BMI and behaviors that may predispose to the development of obesity, such as increased sedentary lifestyle, exposure to unhealthy food advertising, and increased consumption of snacks and energy-dense foods [109].

Although the studies examined provide relevant evidence on the relationship between social media use, neurocognitive development and eating behaviors in adolescence, it is not possible to draw definite and generalizable conclusions as the available data still appear fragmented and heterogeneous from a methodological point of view, especially when based on self-assessments.

Another important aspect to consider is recent evidence regarding the impact of smartphone and other devices use during meals. Emerging studies indicate that this practice has negative consequences both when screens are used by children and when they are used by parents. In particular, parental phone use during mealtimes has been associated with less responsive feeding interactions and less healthy eating practices, potentially compromising children’s self-regulation and family eating routines [112,113]. In addition, parental screen use and the presence of screens during family meals were found to be associated with an increase in both total screen time and problematic social media use among adolescents [114]. Evidence on children’s direct screen exposure confirms these detrimental effects, showing that such behavior disrupts eating practices, reduces emotional interaction, and may hinder child development [115]. Moreover, data on screen exposure during meals among preschool children are already worrying, with studies showing that more than half of preschoolers are affected [116]. The consequences observed are increased overall screen exposure time, more frequent consumption of high-calorie foods and sugary drinks, and less family interaction during meals. Other research on general screen exposure (including during meals) in the same age group has shown that the more time spent on this practice, the lower the development scores (motor skills, language, social skills) and the higher the consumption of UPFs [117]. With regard to adolescents, a very recent cross-sectional study of more than 800 Spanish adolescents showed that using a phone or social networks during meals was the most harmful distraction factor for eating behaviors. Indeed, it was associated with higher consumption of UPFs and lower adherence to the Mediterranean diet, probably due to the characteristics of smartphones, their interactivity and high engagement, which reduce food awareness and family interaction [118].

However, the available studies are still few and mainly observational or based on self-reporting, so more controlled and longitudinal research designs are needed to clarify the causal mechanisms linking screen exposure, family dynamics and eating behaviors.

## 6. When Sharing Becomes Education: The Constructive Side of Digital Media in Food Communication

The context of communication has changed radically. Analysis of the scientific literature has shown that, globally, 5.1 billion people use social media, with the average time spent on these platforms growing from 90 min in 2012 to 143 min in 2024 [119]. This change probably necessitates a renewal of scientific communication strategies: content that traditionally remained confined to books or international scientific journals could therefore be conveyed directly to smartphone screens, making scientific knowledge more accessible and immediately usable by a wider audience. As early as 2016, Campbell et al. had already explored the use of social media by early adopters in healthcare. The results indicated numerous advantages, including patient education and personal development. However, the study also identified the presence of highly generic guidelines that primarily focus on “professionalism,” offering no practical guidance on the optimal methods of online communication [120]. A few years later, the American Academy of Pediatrics once again emphasized the value of social media as an educational tool for both healthcare professionals and families, while recognizing its potential to expand access to care and strengthen patient advocacy practices [121]. The document also highlights other potential applications of social media, such as early detection of risky behaviors, promotion of research, consolidation of professional networking, and provision of online support, despite the critical issues related to privacy protection and the definition of relational boundaries between doctor and patient [121]. This policy statement represents an evolution of previous recommendations, not only addressing the ethical implications of social media use by healthcare professionals but also formulating more concrete recommendations for specific use in pediatrics [121]. This is followed by the very recent position paper of the North American Society of Pediatric Gastroenterology, Hepatology and Nutrition (NASPGHAN) [122], which to date can be regarded as an initial reference point since it provides official and practical recommendations on the use of social media, although it does not yet establish structured and binding rules, as advocated by several researchers [119]. The document calls for greater active participation on social media by healthcare professionals, researchers and institutions, emphasizing that the primary objective is not only to counter misinformation, but also to promote patient advocacy and to disseminate high-quality educational content and research. NASPGHAN further highlights the need to train professionals in the effective use of social media, tailoring language to the target audience (parents, patients, other professionals) and employing images and videos while respecting privacy and copyright, in order to foster constructive interaction. An innovative aspect is the proposal that high-quality social media activities, such as viral educational campaigns or the production of evidence-based content with broad impact, may be recognized as official contributions in the evaluation of promotions or career advancement. In this sense, NASPGHAN acknowledges digital dissemination as an integral component of modern medicine [122]. In this framework, a recent analysis of the role of social media regarding academic communication in pediatric medicine [123] has shown that, although around 70% of physicians have an online presence, few use social media strategically, thus limiting the dissemination of scientific results. Added to this is the transition from chronological timelines (content shown in chronological order, i.e., the most recent posts appeared first) to algorithm-driven feeds, which has made the visibility of scientific content dependent on engagement. This does not promote the quality of information, and in fact, academic metrics (citations, downloads) show little correlation with popularity on social media. However, the evidence analyzed supports the idea that conscious and targeted use of social media can improve scientific dissemination, public engagement and even clinical practice. This has prompted the authors to create an operational framework for the effective use of social media in academia, based on the creation of optimized content, attention to publication timing, ethical collaborations and professional networking, monitoring and documentation of digital impact, but above all, compliance with ethical standards and information quality [123].

Consistent with this, a 2020 systematic review [124] highlighted how well-designed and professionally managed digital tools can have positive effects in both the prenatal and postpartum periods for adolescents and young mothers. The first study on the effects of a large-scale social media intervention on perceptions, attitudes, and key behaviors related to breastfeeding was conducted by Sanchez et al. [125] within the federal Women, Infants, and Children (WIC) program. As part of the study, official Facebook and Instagram pages and campaigns were created and managed using text, images, and videos. The goal was to promote awareness, confidence, self-efficacy, and perceived support for breastfeeding among WIC participants. The results showed an increase in awareness, visits and engagement on the site, a more positive attitude and greater self-efficacy, along with an increase in perceived social support. However, no direct increase in the initiation or duration of breastfeeding was found. This led the authors to conclude that social media-based interventions can amplify some factors critical to breastfeeding success (attitude, self-efficacy and perceived support), although changing actual behaviors identified in breastfeeding initiation and duration is complex and may require more structured and integrated strategies. However, further studies are needed to obtain more robust evidence given the lack of randomization, imprecise measurement of exposure (estimated based on participants’ responses) and the limited duration of the study [125].

A 2023 study [126], although small in size but including different ethnic groups, aimed to explore the factors influencing feeding practices in the first years of life and found that mothers do not only use institutional sources but that there is widespread use of other platforms such as Facebook and Google and that popular consensus becomes an alternative criterion of trust to scientific authority. These results suggest that, for good social communication, it is necessary to enter the digital spaces that parents already frequent with languages and formats that they find familiar in order to combine scientific credibility with the emotional closeness typical of peer groups [126]. Similar results were found in a study [127] analyzing the content of recipes posted in Facebook groups for parents in Thailand. The results showed that Facebook groups are a widely used source of ideas for child nutrition, although the content is not always nutritionally appropriate. Likewise, in this setting the importance of improving social communication emerges, combining visual appeal with adherence to guidelines, possibly with moderation by professionals [127]. The importance of good social communication is further highlighted by a very recent study [128] describing how the Internet and social media are used to search for information on children’s health and growth. The results showed that 70.6% of parents say they search online for information related to their children’s health and growth, and 34% do so frequently (at least once a week), a trend that is even more pronounced among parents of children under two years of age. Social media platforms are the preferred source of information, with Instagram at 31%, Facebook at 30%, YouTube at 22%, and TikTok at 12%. The most alarming finding is that only 33% follow official pages of scientific societies, professional associations, or health ministries, supporting a preference for non-institutional content. Another worrying finding is the verification of information: only 26% say they always check the reliability of the source, 39% do so only occasionally, while 34% never check, further supporting the need for healthcare professionals to guide information through these channels [128].

On the other hand, the first encouraging data are emerging regarding the effectiveness of good communication via social media aimed directly at children and adolescents [129,130]. A systematic review [129] that analyzed the effectiveness and use of social media (e.g., Facebook, Instagram, Twitter) to change diet and/or physical activity in individuals ≥13 years of age, including adolescents, young adults, and adults, showed positive results. Several studies included in the review reported improvements in both dietary behaviors (increased fruit/vegetable consumption and reduced intake of snacks/sugary drinks) and body composition, often accompanied by increases in physical activity. These results led the authors to hypothesize the effectiveness of commercial social media in supporting positive changes in dietary behaviors, although further research with rigorous designs, larger samples, and standardized measurements of engagement and outcomes is needed [129]. Encouraging results also emerge from a recent scoping review [130] mapping the use of social media for nutrition education interventions targeting children aged 10 to 18, which found improvements in weight/BMI and self-reported dietary behaviors, such as increased fruit and vegetable intake. The best outcomes were associated with practical content, peer support, personalization, and family/community involvement, and in some cases, improvements were observed even with low participation. The authors conclude that social media is useful as a platform for nutritional interventions in adolescents, but standardized measurements, cost analysis, and feasibility studies are needed for large-scale implementation [130].

It therefore emerges, as outlined in a very recent work by Raeside [131], that the promotion of adolescent health in the digital age must be based on a participatory and inclusive approach, directly involving young people in the co-design of communication tools without neglecting the digital determinants of health, i.e., factors such as access to technology, algorithms, digital inequalities and exposure to unhealthy content. In this perspective, a recent study has highlighted how health information orientation (personal attitude and motivation in seeking, using and trusting health information) and health literacy are among the main predictors of health-promoting behaviours in adolescents. These findings led the authors to suggest that school and community programmes aimed at enhancing these skills (including the ability to search for, understand and critically evaluate health information found online) may promote more proactive attitudes and healthier behavioural choices [132].

The literature review highlighted the widespread use of non-institutional sources, exacerbated by the dissemination of unverified content on social media, which underscores not only the urgent need for greater active involvement of healthcare professionals in digital contexts, but also the importance of strengthening the digital and health literacy of both parents and adolescents. However, in the meantime, there is growing evidence in favour of the constructive use of social media in health promotion and nutrition education, particularly in relation to children’s health and when digital strategies are designed together with younger users and guided by evidence-based communication principles. It should be noted, however, that the existing literature remains largely descriptive, with few studies providing solid experimental data or long-term follow-up. Moreover, the lack of standardized metrics for assessing the impact of digital communications limits the ability to compare results and accurately measure the effectiveness of online initiatives. 

**Figure 4:**
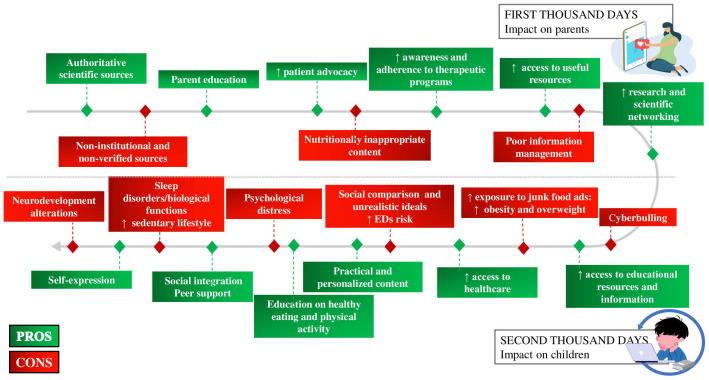
Positive and negative effects of social media on nutrition during the first thousand days (parents) and the second thousand days (adolescents). Abbreviations: ↑ increase; PROS, positive effects; CONS, negative effects.

Figure 4 summarizes the positive and negative effects of social media use in the field of nutrition, distinguishing between the first 1000 days of life, with particular reference to the role of parents, but also to support for scientific progress, promoting advocacy and networking between professionals and institutions, and the second 1000 days, i.e., adolescence.

## 7. Conclusions

Nutritional issues affecting children and adolescents are complex and multifactorial, involving biological, psychological, environmental, and cultural factors. Parenting practices, the environment, and the digital context play a crucial role in shaping attitudes toward food. Early parental influence through inappropriate, overly coercive, or overly permissive parenting styles may contribute to dysfunctional eating patterns, difficulties in self-regulation, and compensatory behaviors over time. In this framework, early and multidisciplinary intervention by pediatricians, psychologists, neuropsychiatrists, and nutritionists is essential to design preventive and support strategies not only for EDs but also to reduce the long-term risk of chronic non-communicable diseases. Future research should focus on validating updated, evidence-based protocols that promote good nutrition from the earliest stages of development, encouraging balance, body awareness through the join efforts of families, schools, healthcare professionals and society. Media education should also be part of this educational synergy, as part of nutritional prevention, helping young people and parents to decode digital content and develop a healthy relationship with body image and nutrition, with a view to a future in which the nutritional health of young people is protected, supported and valued. In fact, the growing exposure of children and adolescents to social media represents a new area of vulnerability, where ambiguous messages about the body, diet and food can contribute to perceptual distortions and dysfunctional habits that add to the misinformation that parents rely on in social media. Therefore, specific guidelines for professionals engaged in scientific dissemination are increasingly needed to ensure that digital communication in the field of nutrition is accurate, ethical and effective.

Finally, it should be noted that most of the available evidence derives from observational and cross-sectional studies, often based on self-reported data. This methodological heterogeneity limits the ability to establish causal relationships and to generalize the findings to different populations and cultural contexts. Future research should therefore adopt more rigorous and standardized designs, including longitudinal and experimental studies, to better define the mechanisms linking social media use, parental behavior, and children’s nutritional outcomes.

Although this narrative review does not represent a quantitative summary of the data, it can provide an up-to-date overview that is useful for future research and preventive strategies.

## Data Availability

Not applicable.
